# Reconstructing intragranular strain fields in polycrystalline materials from scanning 3DXRD data

**DOI:** 10.1107/S1600576720001016

**Published:** 2020-02-21

**Authors:** N. Axel Henningsson, Stephen A. Hall, Jonathan P. Wright, Johan Hektor

**Affiliations:** a Division of Solid Mechanics, Lund University, Box 118, 221 00 Lund, Sweden; b European Synchrotron Radiation Facility (ESRF), 71 Avenue des Martyrs, 38000 Grenoble, France; c Deutsches Elektronen-Synchrotron (DESY), Notkestrasse 85, 22607 Hamburg, Germany; d Helmholtz-Zentrum Geesthacht, Notkestrasse 85, 22607 Hamburg, Germany

**Keywords:** intragranular strain, X-ray diffraction, 3DXRD, tomography

## Abstract

Two methods for reconstructing intragranular strain fields are developed for scanning three-dimensional X-ray diffraction (3DXRD). The methods are compared with a third approach where voxels are reconstructed independently of their neighbours [Hayashi, Setoyama & Seno (2017). *Mater. Sci. Forum*, **905**, 157–164]. The 3D strain field of a tin grain, located within a sample of approximately 70 grains, is analysed and compared across reconstruction methods. Implicit assumptions of sub-problem independence, made in the independent voxel reconstruction method, are demonstrated to introduce bias and reduce reconstruction accuracy. It is verified that the two proposed methods remedy these problems by taking the spatial properties of the inverse problem into account. Improvements in reconstruction quality achieved by the two proposed methods are further supported by reconstructions using synthetic diffraction data.

## Abstract 2

Methods for reconstruction of intragranular strain tensor fields for scanning three-dimensional X-ray diffraction (3DXRD) are developed and evaluated.

## 1. Introduction   

Modern synchrotrons provide X-ray beams of sufficiently high brilliance to enable the study of granular and inter-granular phenomena in dense polycrystalline materials. Relying on the use of parallel and monochromatic X-rays, Poulsen (2004 ▸) and co-workers developed three-dimensional X-ray diffraction (3DXRD). The 3DXRD technique provides a nondestructive way of studying polycrystalline materials on a grain-by-grain basis. Since then, the method has been refined and adopted in several synchrotron facilities across the globe.

In 3DXRD, to avoid diffraction spot overlap, the beam cross section can be reduced, thus limiting the number of simultaneously illuminated grains. The sample must then be rotated and translated to multiple positions to cover a full volume, a procedure which is sometimes known under the name of scanning 3DXRD (Hayashi *et al.*, 2015 ▸). If the beam cross section is small enough, diffraction originating from sub-parts of grains is measured. This opens up the possibility to reconstruct intragranular variations in the crystal structure.

For near-field 3DXRD measurements, using a line beam, suggestions on intragranular orientation reconstructions were first put forth by Rodek *et al.* (2007 ▸), as an extension to previous work on discrete grain mapping (Alpers *et al.*, 2006 ▸). The method was refined by Kulshreshth *et al.* (2009 ▸) to provide access to the intragranular orientation map on a per-voxel basis. None of this work, however, considers intragranular strain, and although it is well known that grain average strain can be determined from far-field 3DXRD measurements, only recently has scanning 3DXRD been used to retrieve intragranular strain variations (Hayashi *et al.*, 2017 ▸; Hektor *et al.*, 2019 ▸). The work of Hayashi *et al.* (2017 ▸) is the first suggestion on how to perform the reconstruction of intragranular strain variations from far-field measurements. The method refines the crystal structure at every point by fitting the orientation and lattice parameters of a single crystal to the subset of reflections that illuminate the point. However, several problems exist with this approach; we have found it may produce artefacts related to both strain state and grain orientation (Henningsson, 2019 ▸).

It has also been suggested, in the case of powder diffraction, that the full strain tensor can be retrieved using filtered back projection with a sufficient number of measurement directions (Lionheart & Withers, 2015 ▸). Similar ideas could, perhaps, be applied to scanning 3DXRD, which measures discrete diffraction events rather than powder rings. If the full strain tensor is to be retrieved via back projection, it would seem that rotations about several different axes are necessary. The time constraints, which are already severe for 3D scanning methods, make such a technique unfeasible. Instead, this paper explores reconstruction techniques that utilize information gathered from rotations about a single axis. As pointed out by Hendriks *et al.* (2019 ▸), the information gathered from rotations about a single axis might be enough to accurately reconstruct the strain distribution. We will present two methods that are capable of reconstructing an intragranular strain tensor field from scanning 3DXRD data. We compare our results with an implementation of the approach suggested by Hayashi *et al.* (2017 ▸) and show how our developments improve the quality of the reconstruction.

Sections 2 and 3 describe the experimental setup and data preprocessing. The frameworks for all three reconstruction approaches are then presented in sections 4, 5 and 6. Reconstructions of the 3D strain field present in a columnar Sn grain embedded in a polycrystalline sample [data originating from the study of Hektor *et al.* (2019 ▸)], together with reconstructions from synthetic diffraction data and error analysis, are presented in Section 7. Finally, the results and their implications are discussed in Section 8.

## 2. Experimental setup   

**Figure 1:**
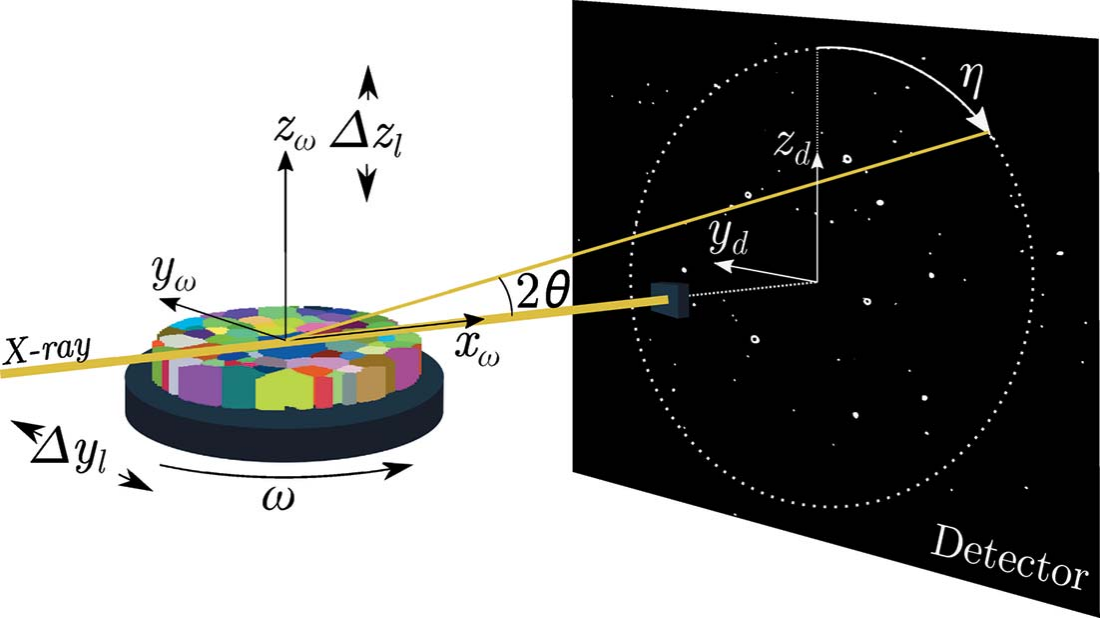
Experimental setup of scanning 3DXRD. The ω turntable holding the sample is rotated around 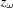 and translated in the fixed *y*_l_ *z*_l_ plane during acquisition.

For scanning 3DXRD, a sample is mounted on an ω turntable that carries a rigidly attached sample coordinate system, subscripted ω (Fig. 1 ▸).

The sample coordinate system is associated with a laboratory coordinate system, subscripted l, which serves as a fixed reference point in all measurements. Both of these coordinate systems are Cartesian, and the 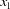 axis is taken as parallel with the incident X-ray beam. During acquisition, the turntable holding the sample is free to rotate around the 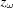 axis and to translate along the fixed transverse beam directions 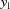 and 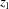. For alignment, the turntable has the freedom to translate in three dimensions, (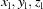), as well as to rotate around each of the three axes (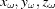). Initially, when no motors of the turntable have been used, the laboratory and sample coordinate systems are by definition aligned. As the detector, situated a distance *D* from the sample, will in general not be mounted perfectly perpendicular to the incoming X-ray beam, an initial calibration of detector tilt and distance is needed. The detector tilt in relation to 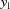 and 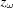, as well as the wedge angle between 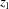 and 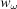, was calibrated following the procedure described in the documentation of the software package *ImageD11* (Wright, 2005 ▸). For further discussion see *e.g.* Oddershede *et al.* (2010 ▸) and Borbely *et al.* (2014 ▸). The intersection between beam centre and detector forms the origin of the 2D Cartesian coordinate system 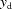
–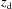. The relation between a vector, 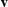, in the laboratory coordinate system and in the sample system now becomes 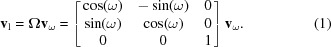
Defining η as the azimuthal angle measured from 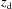 to a considered diffraction peak, the geometry of Fig. 1 ▸ gives the scattering vector, 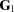, in the laboratory frame as 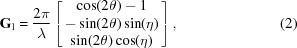
where λ is the X-ray wavelength and θ the Bragg scattering angle. On the basis of the conventions of Busing & Levy (1967 ▸) together with the modified definitions given by Lauridsen *et al.* (2001 ▸), the transformation of a scattering vector from reciprocal space, subscripted *hkl*, to the laboratory frame is 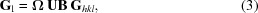
where the columns of the 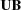 matrix are the reciprocal space lattice vectors. Note that in equation (3), in contrast to Lauridsen *et al.* (2001 ▸), we refer here to a point within a grain rather than the grain average properties, similarly to the work of Alpers *et al.* (2006 ▸). Furthermore, to avoid confusion, it should be noted that, in the work of Lauridsen *et al.* (2001 ▸), an additional coordinate system is used, allowing the sample coordinate system to not be aligned with the ω coordinate system. In our formulation, however, we have taken these coordinate systems to be aligned, and thus the ω system and sample system are one and the same thing. Naturally, the choice of coordinate systems is arbitrary, as long as the transformation operation into the laboratory system is known.

To acquire information on an intragranular length scale the X-ray beam must not illuminate the entire grain during diffraction. The spatial resolution will be limited by the X-ray beam size and the number of different angular projections that can be recorded. To collect data from the entire volume of interest, the turntable, which holds the sample, is translated across the X-ray beam. This means that the rotation axis, 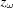, is given a new position in relation to the laboratory coordinate system. At each position the sample is rotated continuously about the 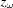 axis and images are integrated and read out every dω degrees. The recorded 2D diffraction pattern at each *y*_l_, *z*_l_, ω setting is the integrated intensity measured over the step length, dω. In the following we refer to the collection of frames taken over a range of ω but at a single *y*_l_, *z*_l_ setting as a ‘frame-stack’. A point in the frame-stack is defined either by the three diffraction angles (η, θ, ω) of Fig. 1 ▸ or by use of the detector plane coordinates (*y*_d_, *z*_d_, ω). The dimensionality of the complete data set is 5D (sample stage position *y*_l_, *z*_l_, ω and diffraction angles η, θ).

## 3. Data preprocessing   

Before strain reconstruction can take place, the 2D diffraction patterns need to be processed to determine the average properties of the grains. The following four steps of analysis summarize the preprocessing:

(1) Image processing: spatial corrections, background subtraction, thresholding and peak centre-of-mass extraction.

(2) Calibration of experimental geometry and determination of scattering vectors 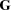.

(3) Peak/grain indexing.

(4) Grain shape reconstruction.

Because the experimental data originated from a FReLoN4M detector, spatial corrections are necessary. These were performed using a dedicated lookup table provided by the ESRF ID11 beamline [see Borbely *et al.* (2014 ▸) for further discussion]. Background correction was then performed, for each frame-stack, on a per-pixel basis, such that for each individual pixel the minimum intensity recorded by the pixel, throughout the frame-stack, was subtracted.

To calculate peak centre-of-mass coordinates, the frame-stack was thresholded and analysed as a volume. Each diffraction peak was extended to a 3D object and assigned the *y*_d_, *z*_d_, ω coordinates of the centre of mass of the 3D intensity distribution. Scattering vectors and Bragg angles can be deduced from the peak centre of mass, after calibration of the experimental setup.

Peak/grain indexing is the procedure to find a set of crystallographic orientations, strains and grain centroid positions that together can correctly account for the observed diffraction data. Grains were indexed using the indexing algorithm in *ImageD11*. To fit an average set of unit-cell parameters to individual grains, methods analogous to those of Oddershede *et al.* (2010 ▸) and Edmiston *et al.* (2011 ▸) were used.

There are several ways to reconstruct the grain shapes from the diffraction data. In this paper we have used filtered back projection, as described by Poulsen & Schmidt (2003 ▸). The sample volume is reconstructed by computing one slice in *z*_l_ at a time and forming, for each grain, a sinogram of diffracted intensities. The inverse Radon transform of the sinogram provides an approximation of the grain shapes and location in the slice. To define grain boundaries, each grain shape was thresholded using a threshold proportional to the most intense voxel within the grain. Overlap between grains was resolved by selecting the grain with the highest intensity at each conflict voxel as the occupant of that voxel. Note that discrete reconstruction methods could provide higher-quality grain maps (*cf*. Alpers *et al.*, 2006 ▸; Rodek *et al.*, 2007 ▸; Kulshreshth *et al.*, 2009 ▸). In this paper, however, we had access to a high number of reflections per grain (>100), and thus the filtered back projection approach performed satisfactorily.

In summary, after preprocessing the diffraction data, we are left with

(1) a list of peak positions (*y*_d_, *z*_d_, ω) with corresponding sample stage (*y*_l_, *z*_l_) settings;

(2) a list of grain average orientations and strains;

(3) a mapping of diffraction peaks to grains;

(4) a voxelated volume describing the grain shapes.

Assuming that the above quantities are available, we proceed, in sections 4–6 [Sec sec6], to describe three methods for intragranular strain reconstruction. Each of these methods relies on the minimization of a cost function. The starting guess in the minimization procedure is taken as the grain average properties emerging from the preprocessing steps described above.

## 4. Single-crystal refinement (SCR)   

It has previously been suggested by Hayashi *et al.* (2017 ▸) that the lattice state at a point 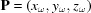 within a grain can be approximated by refining the lattice parameters with respect to the subset of diffraction peaks which intersect 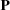. The sample stage translation, Δ*y*_l_, that will ensure that 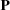 is illuminated at a given ω is found via rotation around the 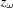 axis: 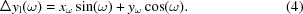
By use of equation (4) the subset of measured diffraction peaks that include scattering from 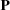 can be extracted. Forward-modelled peak positions, produced using a single-crystal scattering model, are then fitted to the measured peak centre-of-mass coordinates. The resulting lattice orientation and strain tensor are assigned to point 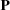.

For a given lattice orientation (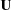), unit cell (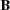) and Miller plane (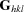), the resulting forward-modelled peak position, expressed in terms of the angles η, θ, ω, is found by combining equations (2) and (3).

In this paper we implement the above concepts, introducing weights to the errors formed between observed and modelled peak positions. The weighted errors (Δη, Δθ, Δω) are taken as 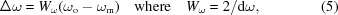


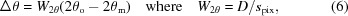


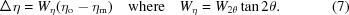
The subscripts o and m stand for observed and modelled, respectively, *D* is the detector-to-sample distance, and *s*_pix_ is the detector pixel size. Note that weighting is essential to account for the experimental resolution being variable with the Bragg angle, θ, and dependent on the selected step size dω.

We assign to 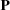 the 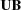 matrix [equation (3)] that minimizes the cost function 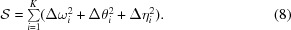
The sum is here taken over all reflections, *K*, that were assigned to 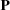 via equation (4). From the resulting optimal 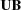 matrix, strain can be computed, given some reference lattice parameters that define a relaxed unit cell.

The minimization of (8) was performed using a least-squares algorithm provided in the Python library SciPy (Jones *et al.*, 2001 ▸). The implementation was based on the *ImageD11* software and can be found at (https://github.com/FABLE-3DXRD/S3DXRD).

The full strain tensor field is retrieved by repeating the single-crystal refinement procedure for all points on a uniform grid with spacing equal to the beam width. As pointed out by Hayashi *et al.* (2015 ▸), the best possible spatial resolution of this approach is limited by double the beam width. This is apparent by considering that equation (4) is fulfilled as long as any part of the beam intersects 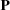.

### 4.1. Inaccuracy and bias   

**Figure 2:**
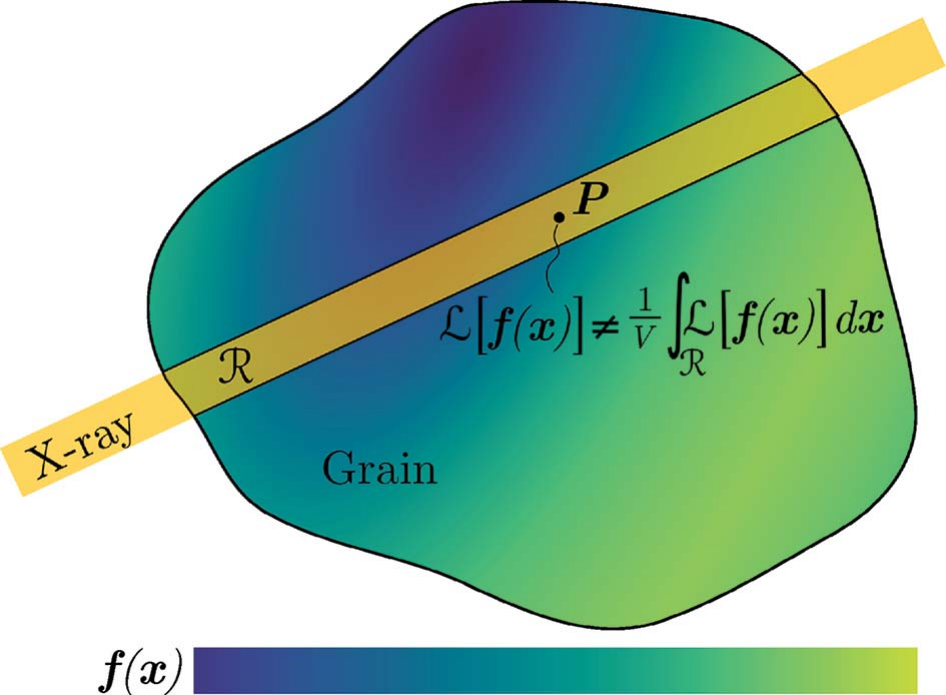
X-ray measurement from a region 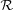 in a grain experiencing a non-uniform vector field, 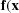), of strain and lattice orientation (illustrated by colour variation). The measured signal from 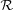 is related to the integral over the intersected region 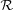, which in general is different from the state at point 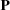.

The key assumption in SCR is that measurements of single points within the volume of a grain can be made. An observed diffraction peak is, however, the result of a volume integral taken over the region of the grain intersected by the beam. The properties of a diffraction peak (η, θ, ω) are therefore average properties, measured over a sub-volume of the grain. In fact, the reconstruction of strain and lattice orientation is a tomography problem, and in general the solution to a ray transform cannot be replaced by a point-by-point fit. By neglecting this fact, SCR will introduce a bias in the reconstructed lattice. Letting the operator 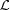 map from the combined strain-orientation field, 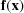, to measurements, and letting *V* denote the volume of an integration region 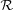, we illustrate the problem in Fig. 2 ▸.

When integration is performed over an illuminated region, the difference between a point measurement, 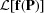, and the integrated value, 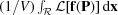, will naturally depend on the distribution of the integrated field. In the case where the integrated field is uniform over the illuminated region, the difference will be zero. If, however, the field varies over the illuminated region, the difference will in general not be zero. If 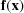 displays sharp features, these will be especially difficult to capture. Likewise, if gradients are present, their magnitudes will in general be reduced, and this damping will be some complicated function of 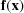 and the distribution of measurements. As we will demonstrate through simulations later (Section 7), the magnitude of these errors can be severe, which motivates the development of new reconstruction methods that respect the tomographic nature of the problem in hand.

## 5. Polycrystal refinement (PCR)   

**Figure 3:**
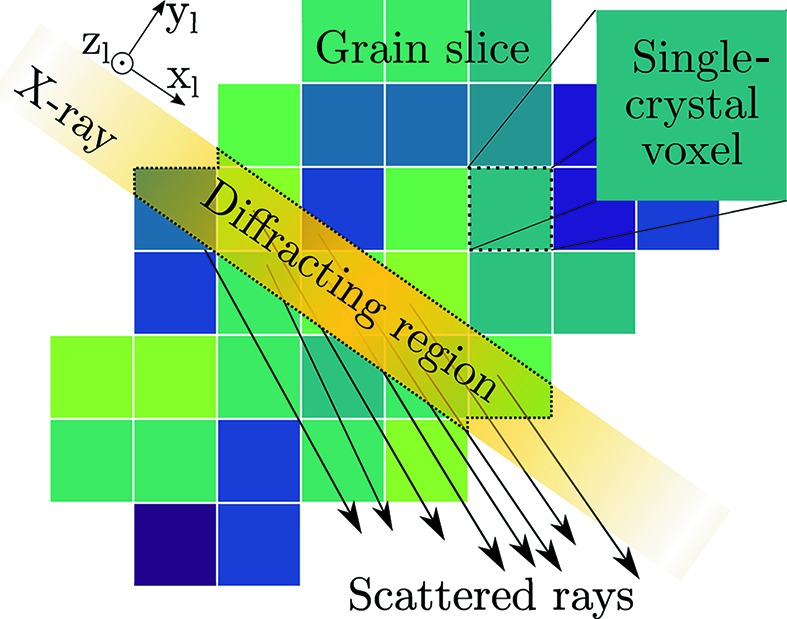
Diffraction is modelled as a set of cubic single crystals. Each crystal carries an independent lattice orientation and strain tensor (illustrated as colour variation).

To remedy the bias of SCR, we seek to formulate a reconstruction method that takes the spatial variation across the grain into account. We propose to discard equation (4) and instead consider all points of the grain simultaneously. This is made possible by modelling diffraction not from one single crystal but from a set of single crystals, similarly to the approach developed by Rodek *et al.* (2007 ▸). Each crystal is made to occupy a discrete voxel within the grain, as illustrated in Fig. 3 ▸.

For a given *y*_l_, *z*_l_, ω setting, all voxels within the grain slice intersected by the beam take part in diffraction. Scattering vectors are assigned using equation (3) and propagated to the detector plane, resulting in clusters of predicted single-crystal diffraction peaks. To form a peak centre-of-mass coordinate from the clustered, simulated, diffraction peaks, the scattered intensities must be taken into account. The intensity scattered from a single crystal is, in general, a function of several variables. However, as the peak centre of mass is sought, only the intensity variation within the peak need be captured. The volume formed by the intersection of beam and voxel is proportional to the number of illuminated unit cells of the single crystal, which in turn is believed to be what dominates intensity variation within a single peak. Using the volume fractions as intensity weights, each peak cluster can be converted to a peak position, (*y*_d_, *z*_d_, ω). More details on the forward model are provided by Henningsson (2019 ▸).

Similarly to SCR, the cost function must be a measure of the mismatch between the observed and modelled diffraction data. Using the Euclidean norm of the peak centre-of-mass coordinates we take the cost as 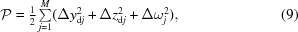
where Δ*y*_d_, Δ*z*_d_ and Δω are the differences between observed and modelled peak positions expressed in units of pixels and rotation step lengths, dω, respectively. The sum in equation (9) ranges over *M*, defined as the total number of observed diffraction peaks of the grain. Notice that no weights with respect to detector position are necessary in (9), as the involved quantities are expressed in units of pixels and rotation step lengths. Instead, in this formulation the modelled and measured diffraction patterns are compared directly using the in-plane detector variables *y*_d_ and *z*_d_ together with the normalized rotation angle ω/dω. Therefore, no discrimination should be made, but all modelled peaks should be considered to fit equally well to the data, as the weighting is already built in to the forward model. Naturally, other factors might also be considered to be included in the weighting, such as photon counts and peak shapes (*cf*. Edmiston *et al.*, 2011 ▸). However, in this work, weighting has been limited to detector positions of the diffraction peaks.

The orientation and strain tensor of each single-crystal voxel composing the reconstructed grain slice is found by minimizing the cost function 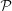. The minimization could be done with respect to Euler angles and lattice parameters, the nine components of the 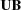 matrix, or Euler angles and the six strain tensor components. In this paper we used the Euler angles and the six strain tensor components. We emphasize that in PCR, like in SCR, the Jacobian of the cost function is determined numerically and the inverse problem is solved by iterative forward modelling. The computational effort of finding the Jacobian can be greatly reduced by using a kinematic approximation such that each voxel scatters independently of its neighbours. This means that the derivative of 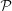 with respect to a single variable, *x*, can be deduced from the current model by replacing only the scattered rays of the voxel affected by the perturbation in *x*. Here the cost (9) was minimized using a standard steepest-descent method (Barzilai & Borwein, 1988 ▸) together with a three-point finite difference scheme.

## 6. Algebraic strain refinement (ASR)   

Polycrystal refinement succeeds in accounting for the spatial dependency of the inverse problem. However, the computational efficiency and complexity of implementation can be improved. Especially desirable would be an easy and efficient implementation of constraints to suppress high-frequency variations in the strain tensor field, emerging from the minimization of equation (9). Such a regularization incorporates the assumption that the strain at a point in the grain is highly correlated to the strain at neighbouring points. To formulate such a method, we drop the concept of a forward model, and instead we seek to find a linear system of equations that will fit a discretized strain-orientation field to diffraction data directly.

In the pursuit of grain average properties, Poulsen *et al.* (2001 ▸) suggested that equation (3) could be used to simultaneously fit strain and orientation for a single grain. In scanning 3DXRD, each measurement provides information on the average scattering vector, 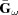, in the region of the grain illuminated by the beam. To accommodate a matrix formulation, linear in the components of the 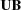 matrix, we recast equation (3) as 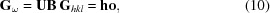
where 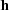 is a 3 × 9 matrix containing the Miller indices (*h*, *k*, *l*) and 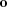 is a 9 × 1 vector that holds the components of the 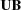 matrix, *UB*_*ij*_, *i.e.*

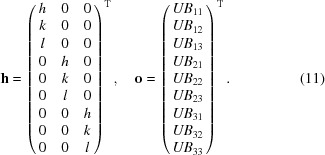
Let us now consider an illuminated region, 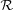, formed by the intersection between beam and grain. Assuming that all points in 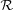 scatter in the rotation interval dω, the average scattering vector becomes 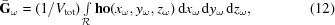
where 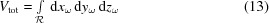
and 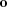 is allowed to vary in 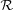. Discretizing the grain into voxels and approximating 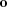 as constant over each voxel, equation (12) gives 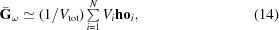
where *N* is the number of voxels and *V*_*i*_ the volume of intersection between 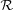 and voxel number *i*. If all observed scattering vectors of a grain are considered simultaneously, a matrix formulation is achieved:
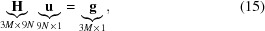
where 
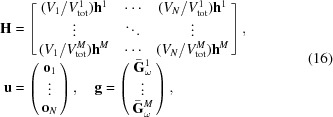
and the total number of measured scattering vectors is *M*. By solving equation (15) in a least-squares sense, the orientation and strain state of the grain can be retrieved. Before doing so, however, the incorporation of weights to account for variable experimental resolution is needed. Additionally, some constraint of smoothness to the strain is required. These seem possible to derive and impose, but not trivial to implement. If the constraints are to be formulated in terms of absolute smoothness of strain, the conversion into 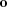 makes them nonlinear. Although methods for solving such problems exist, they seem to scale poorly with the number of unknowns unless the derivatives of the constraints can be provided analytically. Therefore we choose a simpler formulation, where the matrix equation is linear in the strain tensor components directly. This is possible by converting the peak centre-of-mass coordinates into average strain measurements.

### 6.1. Peak position to average strain   

As pointed out by Poulsen *et al.* (2001 ▸) and Margulies *et al.* (2002 ▸), for hard X-rays and small Bragg angles the strain associated with a reflection is well approximated by 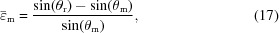
where θ_m_ is the measured angle of diffraction and θ_r_ is the corresponding angle of diffraction expected for a relaxed reference state. The scalar measured strain, 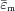, is an average property of the region 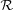, and as explained by Lionheart & Withers (2015 ▸), it exists in the direction perpendicular to the diffracting Miller planes.

**Figure 4:**
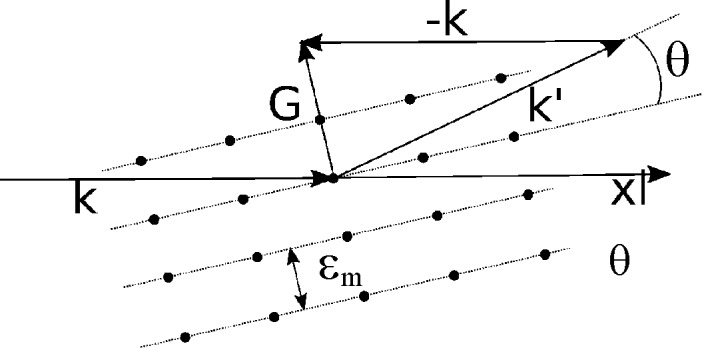
Bragg scattering from a 2D lattice. The direction in which the average strain 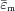 exists is parallel to 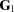.

Considering the definition of the scattering vector, 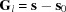, illustrated in Fig. 4 ▸, a unit vector, 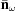, in the strain direction is given as 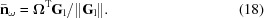
Using equations (17) and (18), each measured peak position can be converted to a corresponding average strain, 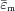, and average strain direction, 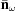. Considering multiple measurements from a single grain, the strain tensor can be deduced from the two quantities (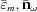), as laid out by Poulsen *et al.* (2001 ▸) and Margulies *et al.* (2002 ▸). In their original work, part of the strain tensor was retrieved as a grain average property. Here, we seek to extend these concepts to the scanning 3DXRD case and compute the full strain tensor field, as it varies spatially within a grain.

### 6.2. Matrix formulation   

In analogy with equation (12), we have 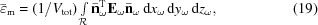
where 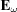 is the strain tensor at point 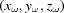 given in the ω coordinate system. The discrete form becomes 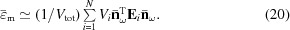
Considering all measured scattering vectors of a grain we can introduce a projection matrix, 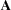, that projects a given strain tensor field, specified by the vector 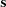, into average strain measurements, 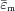. If the measured average strains are stored in the vector 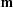, we seek the solution, 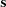, to the linear equation system 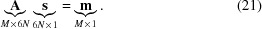
Explicitly, the matrices 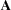, 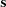 and 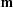 take the form 
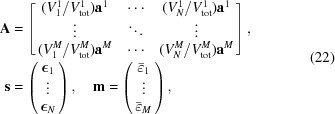
where 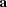 contains the components of the strain direction 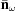 as 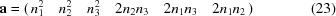
and 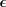 contains the six independent strain components of a voxel in Voigt notation:
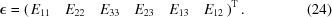



### 6.3. Weighting   

Since equation (21) is formulated in terms of average strain, which is a function of diffraction angle θ, the weights should be related to the measurement uncertainty in θ. For a given measurement we choose here the weight, *w*, as 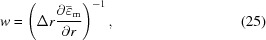
where Δ*r* is the measurement uncertainty in the radial direction of the detector, *r*, and 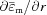 can be found numerically from equation (17). The value of Δ*r* can be extracted from peak-by-peak fits (Edmiston *et al.*, 2011 ▸). In the specific cases presented in this paper, we make a simplification and assume a constant value Δ*r* = 0.1 pixels (Borbely *et al.*, 2014 ▸). This is motivated by a low-angle approximation for high-energy diffraction. In the work presented here, the maximum angle of diffraction was 2θ = 16°. The constant value selected for Δ*r* will have no impact on the weighted solution to the problem. However, if one seeks to evaluate the fit quality of computed strains, 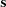, to data, 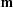, a selection of Δ*r* is necessary to indicate the error margin of the measurements.

In matrix format we now have 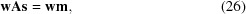
where 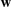 is a diagonal matrix holding the weights.

### 6.4. Constraints   

If the least-squares solution to (26) is sought, the corresponding cost function could be formulated as 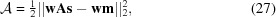
where ||·||_2_ is the Euclidean norm. We formulate the desired smoothness constraint for each component of strain, *E*_*ij*_, as 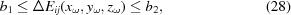
where Δ*E*_*ij*_(*x*_ω_, *y*_ω_, *z*_ω_) is the difference in strain between two neighbouring voxels. The fixed bounds *b*_1_ and *b*_2_ provide a lower and upper bound, respectively, and therefore regulate the maximum change in strain between two voxels. Neighbours are here defined as two voxels in a grain slice that share at least one corner point. The minimization of (27) under the constraint of (28) can be performed in several ways. Here, we have used a trust-region algorithm described by Byrd *et al.* (1999 ▸) and implemented in the Python library SciPy. Whatever iterative scheme is deployed, it is emphasized that both the Jacobian and the Hessian of the problem are known analytically, something which simplifies the minimization of equation (27).

## 7. Results   

**Figure 5:**
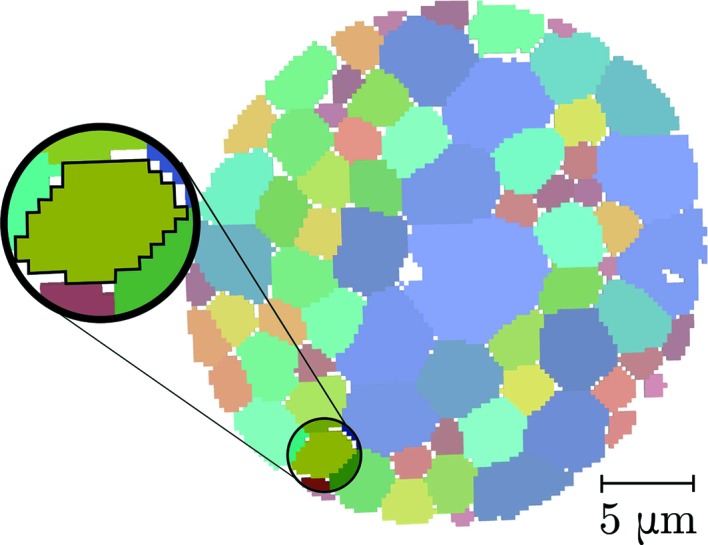
Sample cross section at *z* = 0 of the sample scanned by Hektor *et al.* (2019 ▸), produced via filtered back projection. The grains are randomly coloured by index, with the grain selected for strain reconstruction highlighted. The voxel dimension is 0.25 × 0.25 × 0.25 µm.

The strain state of a columnar tin (Sn) grain was reconstructed with the presented methods: SCR, PCR and ASR. The diffraction data originated from the experiment described by Hektor *et al.* (2019 ▸) and were collected at the nanostation of the ESRF ID11 synchrotron beamline. The grain selected for reconstruction (Fig. 5 ▸) was chosen because it exhibited a strain gradient, found in previous work using the SCR method. In principle, there is no hindrance to performing reconstructions for full sample volumes, featuring many grains. However, the focus of this article is to validate the theory and approximations underlying the presented reconstruction methods. For further practical applications the implementations should be optimized, and we note that when reconstructing many grains simultaneously all three methods are easily run in parallel.

Relevant experimental parameters can be found in Table 1 ▸.

Preprocessing of the diffraction data was performed primarily using the *FABLE* software suite (Sørensen *et al.*, 2012 ▸). The grain shapes were deduced using filtered back projection as discussed in Section 3. Implementation of the back projection is available at https://github.com/FABLE-3DXRD/S3DXRD, together with implementations of the three reconstruction algorithms.

Owing to time constraints, the experiment was performed with a step size of 0.5 µm in *z*, which is to be compared with the beam size of 0.25 µm. Linear interpolation between reconstructed slices has thus been performed in the presentation of 3D strain fields. Further specifics regarding the sample preparation, background of the experiment and diffraction data preprocessing are given by Hektor *et al.* (2019 ▸).

As strain is a measure of relative displacement, a reference configuration must be selected. Here we have used the lattice parameters of Table 2 ▸ to define a relaxed Sn unit cell. These parameters represent the sample average lattice parameters, calibrated during grain indexing using the *ImageD11* software.

**Figure 6:**
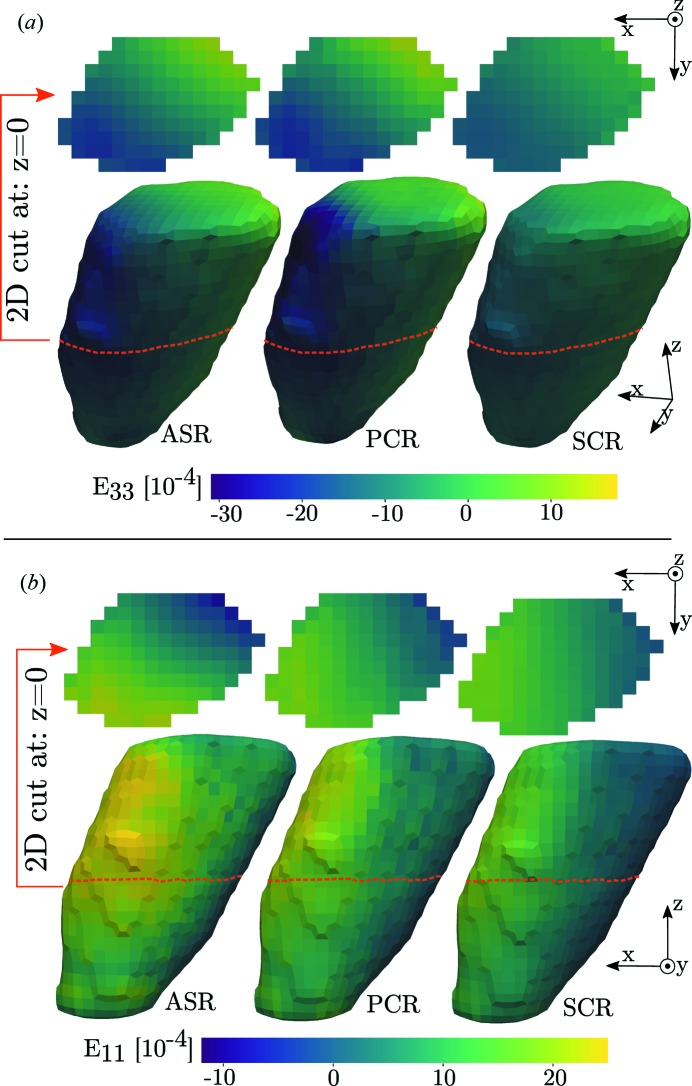
Reconstruction of axial strain tensor components, *E*_33_ in (*a*) and *E*_11_ in (*b*), for a tin grain. The result is seen to vary with reconstruction method from left to right. A smoothing filter has been applied to the topology of the 3D grain surface for visualization purposes. Two-dimensional cross sections, at *z* = 0, are illustrated, with the method varying from left to right.

All strain fields presented in this paper are given in the ω coordinate system. In ASR, the constraint imposed on the strain difference, Δ*E*_*ij*_, between two neighbouring voxels was taken as 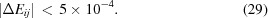
The resulting reconstructions of the selected grain are presented in Fig. 6 ▸.

**Figure 7:**
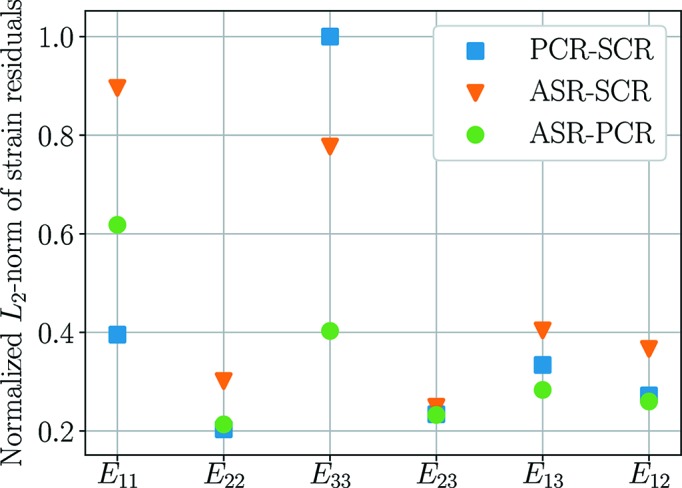
Normalized Euclidean norms of the difference in reconstructed strain compared between SCR, PCR and ASR. PCR-SCR corresponds to taking the solution of SCR at every point and subtracting it from the solution of PCR *etc*. The data correspond to the grain in Fig. 6 ▸.

The agreement between the reconstructions provides important information on the accuracy of the methods. A set of residual fields are introduced to illustrate this. These are defined as the difference in reconstructed strain fields between the three methods. Three such fields can be formed, subtracting the results of SCR from the results of ASR and PCR, and the results of PCR from those of ASR. The Euclidean norms of these residual fields are presented in Fig. 7 ▸ and provide an overview measure of agreement between the three methods.

**Figure 8:**
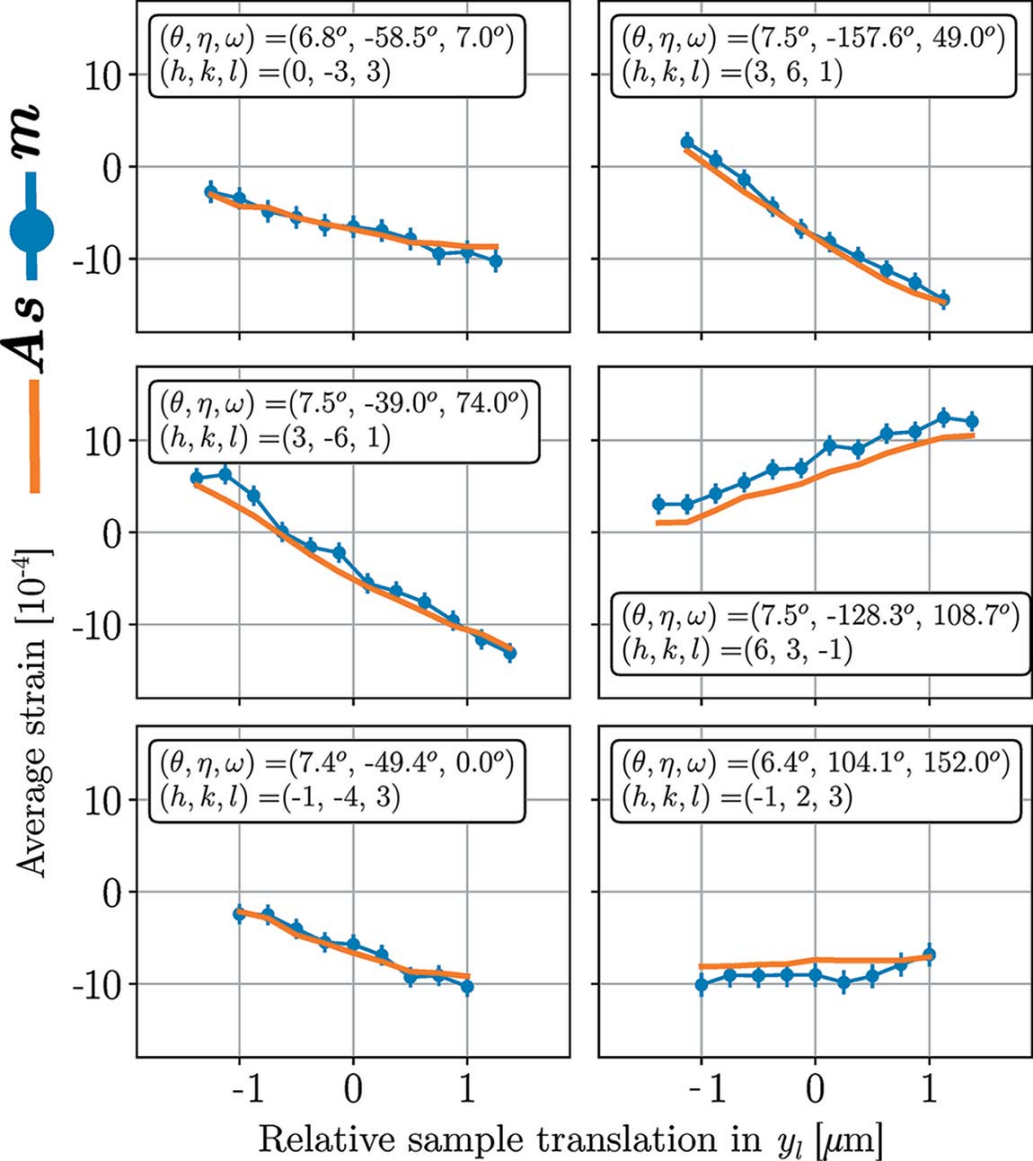
Subsets of computed average strains (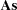) compared with subsets of measured average strains (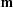) for ASR. Each subplot corresponds to the strain profile of a single selected diffraction peak. This means that different subsets of the scalar instances of the vectors 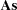 and 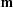 are displayed in the six subplots. The data originate from the grain in Fig. 6 ▸, where a total number of 321 diffraction peaks were used for fitting. The error bars were computed as the reciprocal of the weights in equation (25), with Δ*r* = 0.1.

Regarding ASR, the fit of the solution, **s**, to measurements, **m**, can be evaluated by analysing individual diffraction peaks. Such analysis can also serve as verification that any reconstructed strain gradients are indeed present in the underlying data. In Fig. 8 ▸ the product **As** is plotted against the measured average strains, **m**, for six selected diffraction peaks out of 321 used peaks, at grain slice *z*_l_ = 0. The peaks were selected to give a good spread in η, ω and to have a relatively high diffraction angle, θ, since such peaks have a higher influence (weight) on the solution of the least-squares problem. Each presented diffraction peak is associated with a set of Miller indices (*h*, *k*, *l*) and an angular setting (θ, η, ω), as indicated in the subplots of Fig. 8 ▸. As the grain is translated across the X-ray beam the Miller planes experiencing a favourable Bragg condition will diffract, creating a profile of average strain along the beam. Multiplying the constant uncertainty in peak position, Δ*r*, by the strain sensitivity, 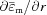, provides an estimate of the local strain uncertainty of each measurement [*i.e.* the inverse of the weights in equation (25)]. To illustrate this, error bars have been put on the measurement points in Fig. 8 ▸. The expected uncertainty was taken as Δ*r* = 0.1 pixels, in accordance with the work of Borbely *et al.* (2014 ▸).

**Figure 9:**
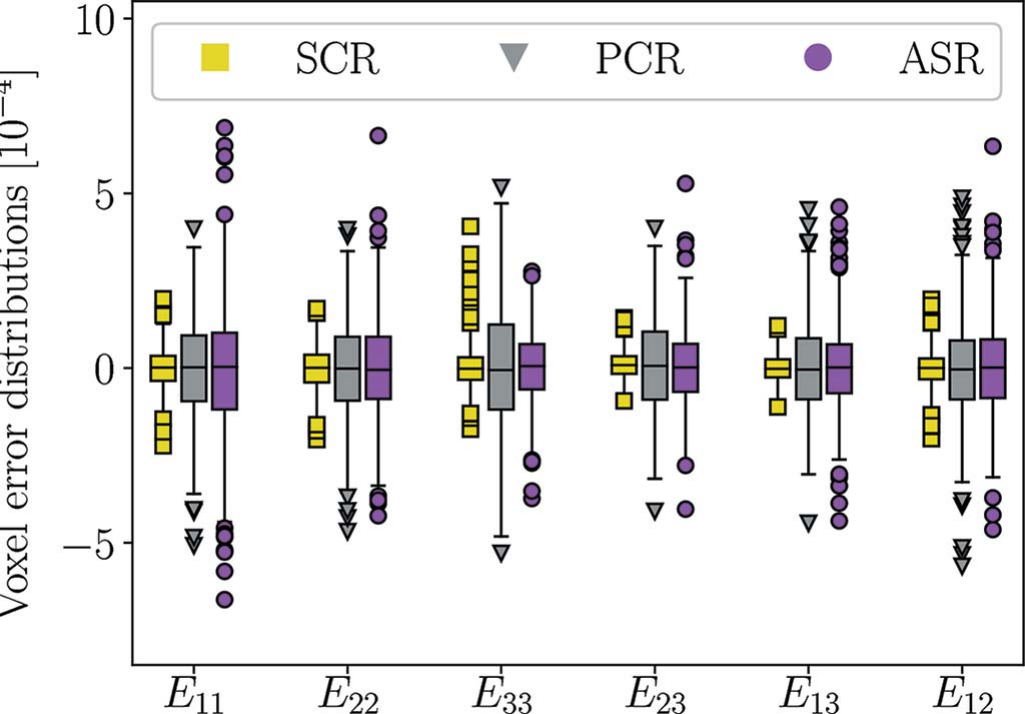
Error in strain fields due to normally distributed noise in peak positions. Outliers are defined by deviations from the mean value by more than ±1.5 times the interquartile range. The data correspond to the grain in Fig. 6 ▸.

To evaluate the impact of noise on the reconstructed strain fields, the peak positions were perturbed and a secondary reconstruction performed. Noise was drawn from a normal distribution with expectation value 0 and standard deviation σ: 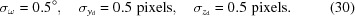
It is important to appreciate that noise is introduced into the peak centre-of-mass coordinates rather than the raw detector images. The peak positions are normally computed by combining several pixel intensities, and thus a given perturbation of the peak position will, in general, correspond to a greater measurement noise in the raw data. However, to investigate the worst-case scenario, when a diffraction peak is composed of a single pixel, we select the noise as stated in equation (30). Residual fields were defined as the difference between reconstructed strain fields using the perturbed and original peak centre-of-mass positions, respectively. An estimate of the propagated error is retrieved by down-sampling the residual fields into 2 × 2 voxel sub-regions. If instead the field is not down-sampled the propagated error will appear greater. However, the resolution of the SCR method is two times the beam width as discussed previously. Furthermore, it is reasonable to define error in terms of low-frequency variations in the strain field. The down-sampled residual fields are presented as a box plot in Fig. 9 ▸, including all three methods and six strain components.

**Figure 10:**
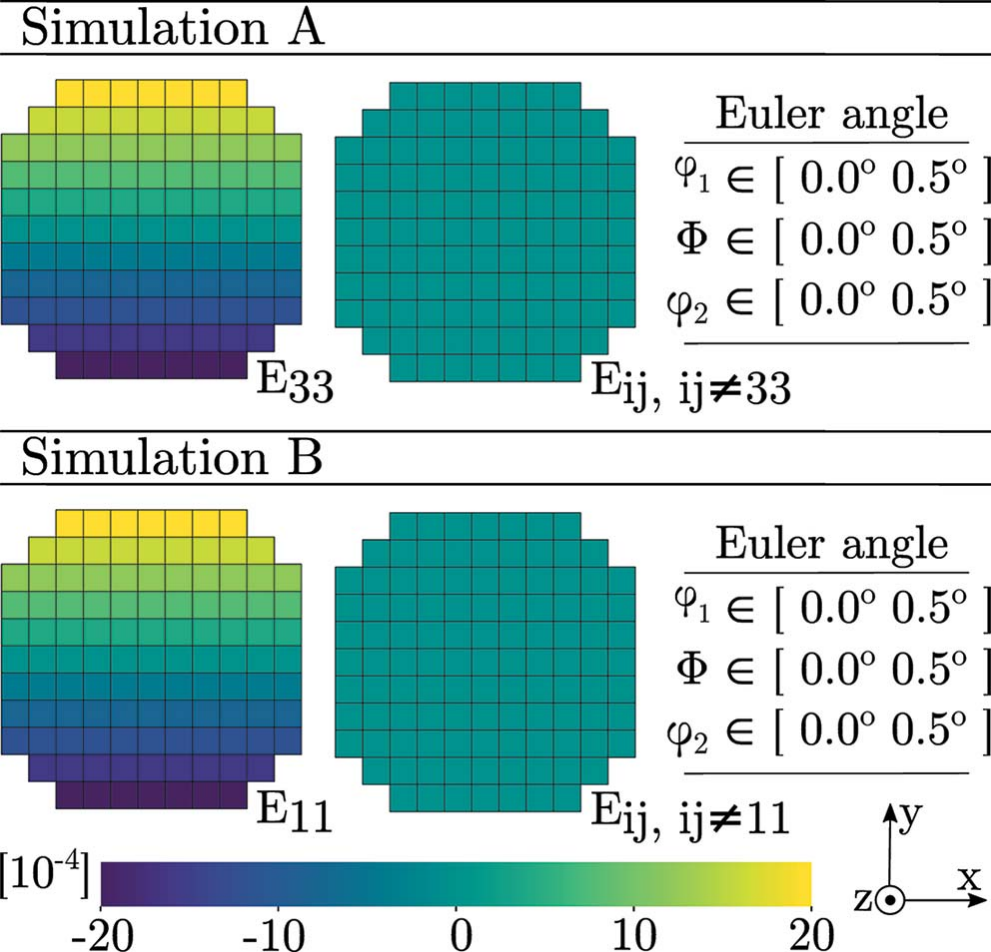
Input topology, strain state and mosaicity of a simulated tin grain slice comprising a total of 109 voxels. In both A and B all three Euler angles (φ_1_, Φ, φ_2_) vary linearly with *x*, from 0° (left) to 0.5° (right).

Finally, we present reconstructions of synthetic diffraction data produced using the forward model coupled to PCR. Before peak centre-of-mass coordinates were computed, the modelled data were binned by dω and detector pixel size. The parameters of the simulations were taken to equal those of Tables 1 ▸ and 2 ▸ and equation (29). Diffraction from an Sn slice was simulated two separate times, featuring a linear strain gradient in either *E*_33_ or *E*_11_. The input to these two simulations is presented in Fig. 10 ▸.

**Figure 11:**
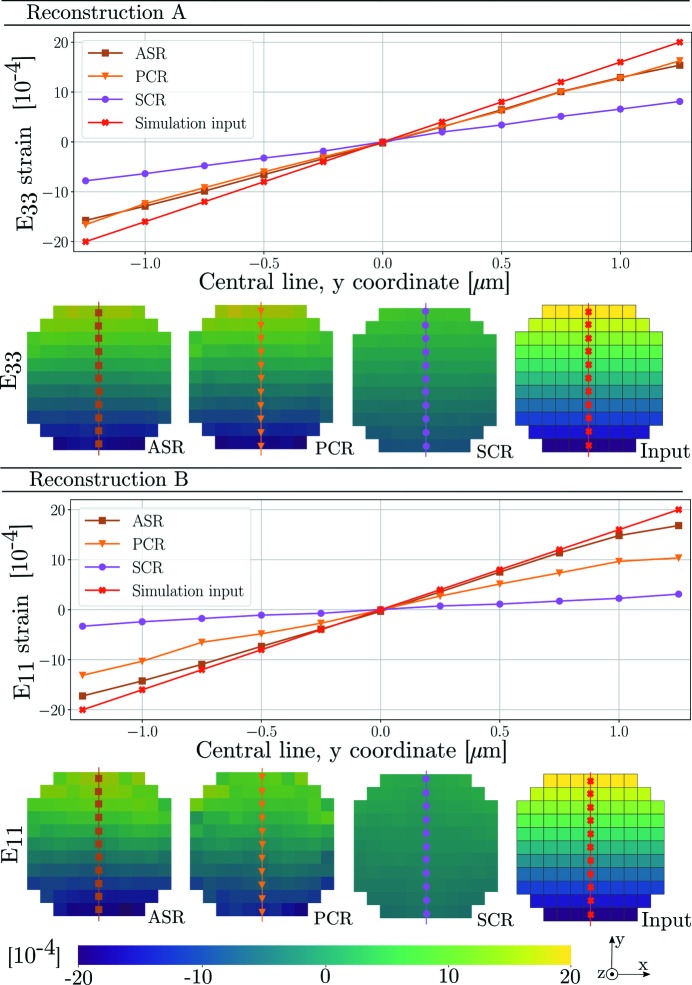
Reconstruction of axial strain tensor components corresponding to the two simulations presented in Fig. 10 ▸. The result is seen to vary with reconstruction method from left to right. The strain along the central vertical line of the grain is illustrated as line plots for each of the simulations.

To mimic a mosaic spread, a gradient in each of the three Euler angles (φ_1_, Φ, φ_2_) was introduced. Starting from a crystal orientation aligned with the ω coordinate system, φ_1_ = Φ = φ_2_ = 0, the gradient was made to increase in the positive *x*_ω_ direction by uniformly increasing all of the Euler angles to a maximum of φ_1_ = Φ = φ_2_ = 0.5°. The results of the two simulations are presented in Fig. 11 ▸.

To investigate the reconstruction of more complex strain states, a third simulation has been performed (Appendix *A* [App appa]). This simulation features strain in all six components of the strain tensor and can thus provide insight into the reconstruction of shear strains, which have not been covered much in the above.

## 8. Discussion   

Fig. 7 ▸ indicates that the greatest discrepancy in reconstructed strain between the three methods is found in the *E*_11_ and *E*_33_ strain components. Turning first to the *E*_33_ strain, we find that ASR and PCR are in agreement while the reconstruction of SCR deviates. Indeed, the 3D reconstructions in Fig. 6 ▸(*a*) reveal reduced amplitudes for SCR. As discussed in Section 4, this is explained by the invalid assumption of sub-problem independence made in SCR. Reflections probing the *E*_33_ strain are available from all ω angles, and thus the single-crystal fit to a point will be influenced by points across the entire grain. Simulation A, presented in Fig. 11 ▸, further implies that ASR and PCR here provide more accurate descriptions of the strain state than SCR.

Regarding the *E*_11_ strain, Fig. 7 ▸ shows a higher level of agreement between PCR and SCR than between ASR and PCR. This is an example of when the assumption of sub-problem independence happens to work. Examining Fig. 6 ▸(*b*) we see a strain gradient with a significant component along the *x* axis. This strain will be probed mostly at ω ≃ 90°, *i.e*. perpendicular to the gradient direction. This means that the single-crystal fit to any point will be influenced mostly by points featuring the same *E*_11_ strain. If instead the *E*_11_ strain state had featured a gradient with a significant component in the *y* direction, SCR would again break down. This is verified by simulation B, also presented in Fig. 11 ▸, where the *E*_11_ strain gradient has been selected to align with the *y* direction instead of the *x* direction.

Apart from the confirmation of bias in SCR, which is related to the direction of the gradient, we also note that simulations A and B imply that the *E*_33_ strain component is more retrievable than the *E*_11_ component for ASR and PCR. To understand this we emphasize that, in general, measurements of a specific strain component are not uniformly sampled. In this case, although the strain in the direction of the rotation axis, *E*_33_, has an equal chance of being sampled at any given ω setting, the strain along the beam direction, *E*_11_, will mostly be probed close to ω = 90°. Therefore, the reconstruction of the *E*_11_ strain will be a less well posed tomography problem than the reconstruction of *E*_33_. This was also noted by Margulies *et al.* (2002 ▸).

The diffraction peak analysis presented in Fig. 8 ▸ verifies that the reconstructed strain gradients are indeed present in the underlying data. In regards to the fit quality of ASR, we draw attention to the use of absolute strains in the reconstruction procedure. If the average position of each diffraction peak had been subtracted before reconstruction, it is possible that some systematic errors could be avoided. However, such a method would unfortunately not be able to give approximations to the absolute values of strain but would be limited to reconstructing relative strain variations within the grains.

The interquartile range of the propagated errors in Fig. 9 ▸ is approximately 2 × 10^−4^ or lower for all three methods. The elevated sensitivity of ASR and PCR compared with SCR is believed to be related to the incorporation of volume weights. In ASR and PCR, few reflections can carry a high weight in relation to a strain component for a voxel. This leads to a diminished probability for noise to cancel out between reflections. In SCR, all reflections related to a voxel are equally weighted in terms of illuminated voxel volume, and thus the perturbations in peak position are more likely to cancel out. This is a necessary deficit of PCR and ASR, as any method taking the spatial dependence of the problem into account must also incorporate some sort of weighting based on illuminated fractions. Therefore, it would seem that the precision of SCR, seen as compact distributions in Fig. 9 ▸, is a symptom of the damping of the strain field.

It should be recognized that the inverse problem being undertaken features coupling between strain and orientation. This means that a strain state can give diffraction peak position shifts not only in 2θ but also in ω and η. PCR aims to recover both orientation and strain, while ASR assumes a uniform orientation within the grain. However, Figs. 11 ▸, 14 and 15 (see Appendix *A* [App appa]) indicate that the input orientation gradient has a small impact on the strain reconstruction of both ASR and PCR. In fact, the strain reconstruction of ASR is more accurate than that of PCR. This is promising as ASR is both computationally faster and easier to implement than PCR.

For further work it could be interesting to incorporate a compatibility or equilibrium constraint into the strain reconstruction, similar to what is suggested by Jidling *et al.* (2018 ▸) and for equilibrium constraints demonstrated through simulations of bulk materials by Hendriks *et al.* (2019 ▸). Such constraints enjoy a simple physical interpretation and would in this sense be a superior choice over the smoothness constraint adopted in ASR. Furthermore, in the case of considerable plastic deformation, when the crystals display abrupt lattice discontinuities, the validity of the smoothness constraint could be questioned.

Additionally, the PCR method suggests that more detailed information in the raw data could be taken into account since the driving model produces synthetic diffraction patterns. For instance, the match between peak shapes could be used instead of peak centre-of-mass coordinates to enhance accuracy. This could be performed by modifying the cost function (9) to incorporate the activated pixel pattern, similarly to Suter *et al.* (2006 ▸) and Li & Suter (2013 ▸).

## 9. Conclusions   

Work towards reconstructing the strain tensor variation on an intragranular level for scanning 3DXRD experiments is presented. It is established that reconstruction methods should take the spatial (tomographic) properties of the inverse problem into account. Through simulations, the PCR and ASR methods developed in this paper have been shown to provide more consistent approximations to the input strain tensor fields than the previously suggested method, SCR. The ASR method operates on the assumption of a smooth strain field and should be used with caution in the presence of lattice discontinuities. The methods have been shown to be computationally viable in the context of synchrotron diffraction data by reconstructions of a tin grain embedded within a polycrystalline sample. By analysing individual diffraction peaks, it was verified that the reconstructed strain gradient was a real feature of the underlying data.

## Figures and Tables

**Figure 12:**
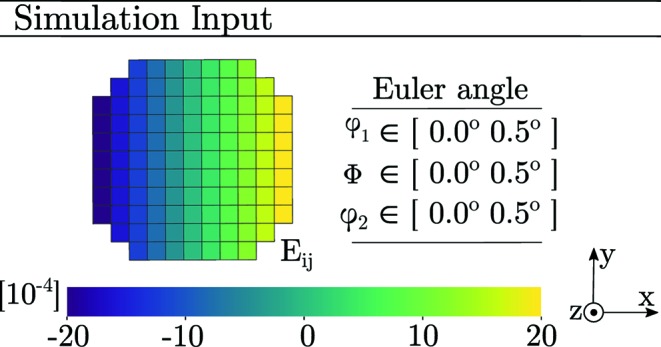
Input topology, strain state and mosaicity of a simulated tin grain slice comprising a total of 109 voxels. All three Euler angles (φ_1_, Φ, φ_2_) vary linearly with *x*, from 0° (left) to 0.5° (right). Likewise all six strain components vary linearly with *x*, from −20 × 10^−4^ (left) to +20 × 10^−4^ (right).

**Figure 13:**
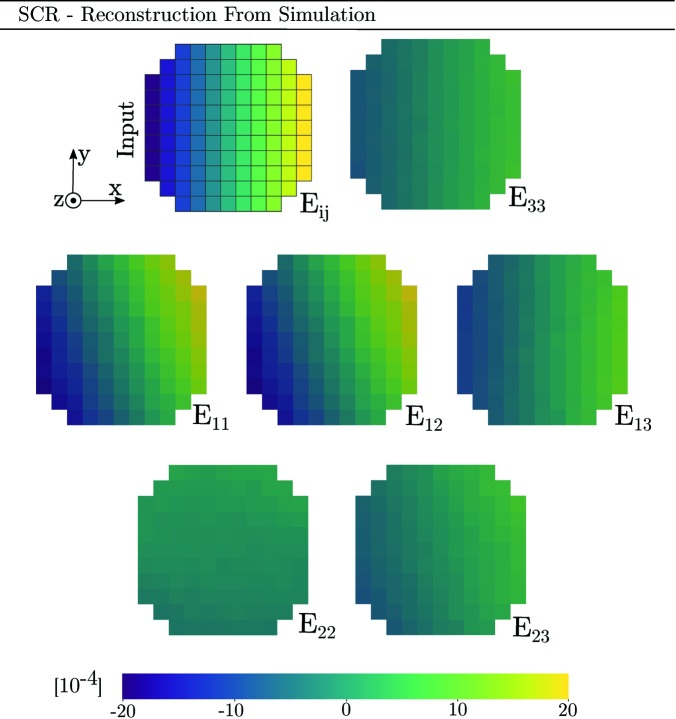
Reconstruction by SCR of full strain tensor corresponding to the simulation presented in Fig. 12 ▸. The top-left sub-figure represents the simulation input strain field, rescaled to the current colour range.

**Figure 14:**
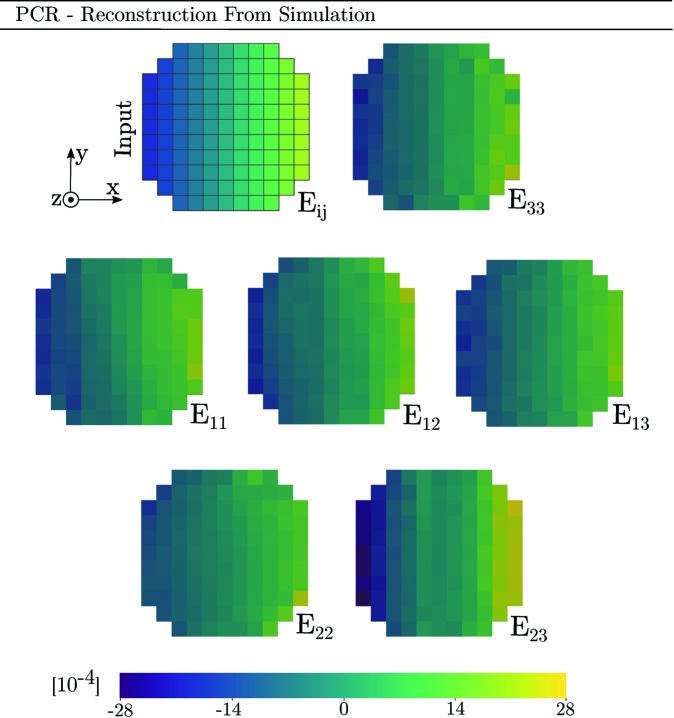
Reconstruction by PCR of full strain tensor corresponding to the simulation presented in Fig. 12 ▸. The top-left sub-figure represents the simulation input strain field, rescaled to the current colour range.

**Figure 15:**
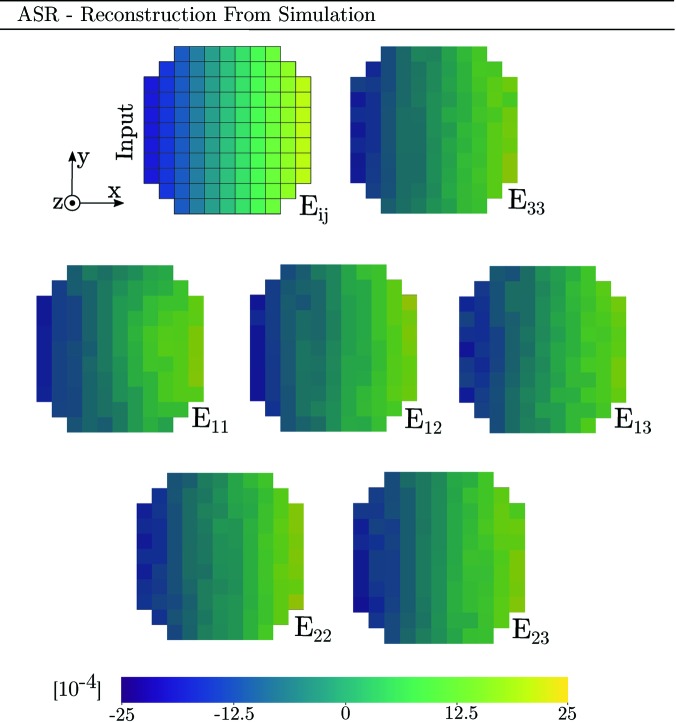
Reconstruction by ASR of full strain tensor corresponding to the simulation presented in Fig. 12 ▸. The top-left sub-figure represents the simulation input strain field, rescaled to the current colour range.

**Table 1:** Experimental parameters

Wavelength	0.22 Å
Sample-to-detector distance	163 mm
Dectector pixel size	50 × 50 µm
Detector dimensions	2048 × 2048 pixels
Beam size	0.25 × 0.25 µm
ω rotation interval	[0°, 180°]
ω step length	1°

**Table 2:** Relaxed reference lattice parameters

*a*	*b*	*c*	α	β	γ
5.81127 Å	5.81127 Å	3.17320 Å	90.0°	90.0°	90.0°

## Appendix A Further reconstructions from synthetic diffraction data

To further investigate the reconstruction quality of the full strain tensor, *E*_*ij*_, an additional simulation and reconstruction set is presented. The simulation was defined similarly to that of Fig. 10 ▸ with two exceptions. (i) Strain gradients were introduced in all six strain tensor components simultaneously. (ii) The linear strain gradients were taken to vary in the *x* direction. The simulation input is illustrated in Fig. 12 ▸. Note that in the corresponding reconstructions of SCR, PCR and ASR (Figs. 13 ▸–15 ▸ ▸) the colour bar is rescaled to facilitate strain values that exceed the simulation input range. Each figure therefore includes a reference input grain slice to enable comparisons.
